# Diagnostic Accuracy of Rapid Antigen Tests for COVID-19 Detection: A Systematic Review With Meta-analysis

**DOI:** 10.3389/fmed.2022.870738

**Published:** 2022-04-07

**Authors:** Maniya Arshadi, Fatemeh Fardsanei, Behnaz Deihim, Zahra Farshadzadeh, Farhad Nikkhahi, Farima Khalili, Giovanni Sotgiu, Amir Hashem Shahidi Bonjar, Rosella Centis, Giovanni Battista Migliori, Mohammad Javad Nasiri, Mehdi Mirsaeidi

**Affiliations:** ^1^Infectious and Tropical Diseases Research Center, Health Research Institute, Ahvaz Jundishapur University of Medical Sciences, Ahvaz, Iran; ^2^Department of Microbiology, School of Medicine, Ahvaz Jundishapur University of Medical Sciences, Ahvaz, Iran; ^3^Medical Microbiology Research Center, Qazvin University of Medical Sciences, Qazvin, Iran; ^4^Department of Bacteriology and Virology, School of Medicine, Dezful University of Medical Sciences, Dezful, Iran; ^5^Department of Microbiology, School of Medicine, Shahid Beheshti University of Medical Sciences, Tehran, Iran; ^6^Istituti Clinici Scientifici Maugeri IRCCS, Tradate, Italy; ^7^Clinician Scientist of Dental Materials and Restorative Dentistry, School of Dentistry, Shahid Beheshti University of Medical Sciences, Tehran, Iran; ^8^Clinical Epidemiology and Medical Statistics Unit, Department of Medical, Surgical and Experimental Sciences, University of Sassari, Sassari, Italy; ^9^Division of Pulmonary and Critical Care, College of Medicine-Jacksonville, University of Florida, Gainesville, FL, United States

**Keywords:** COVID-19, SARS-CoV-2, rapid antigen test, specificity, sensitivity, meta-analysis

## Abstract

**Introduction:**

Reverse transcription-polymerase chain reaction (RT-PCR) to detect SARS-CoV-2 is time-consuming and sometimes not feasible in developing nations. Rapid antigen test (RAT) could decrease the load of diagnosis. However, the efficacy of RAT is yet to be investigated comprehensively. Thus, the current systematic review and meta-analysis were conducted to evaluate the diagnostic accuracy of RAT against RT-PCR methods as the reference standard.

**Methods:**

We searched the MEDLINE/Pubmed and Embase databases for the relevant records. The QUADAS-2 tool was used to assess the quality of the studies. Diagnostic accuracy measures [i.e., sensitivity, specificity, diagnostic odds ratio (DOR), positive likelihood ratios (PLR), negative likelihood ratios (NLR), and the area under the curve (AUC)] were pooled with a random-effects model. All statistical analyses were performed with Meta-DiSc (Version 1.4, Cochrane Colloquium, Barcelona, Spain).

**Results:**

After reviewing retrieved records, we identified 60 studies that met the inclusion criteria. The pooled sensitivity and specificity of the rapid antigen tests against the reference test (the real-time PCR) were 69% (95% CI: 68–70) and 99% (95% CI: 99–99). The PLR, NLR, DOR and the AUC estimates were found to be 72 (95% CI: 44–119), 0.30 (95% CI: 0.26–0.36), 316 (95% CI: 167–590) and 97%, respectively.

**Conclusion:**

The present study indicated that using RAT kits is primarily recommended for the early detection of patients suspected of having COVID-19, particularly in countries with limited resources and laboratory equipment. However, the negative RAT samples may need to be confirmed using molecular tests, mainly when the symptoms of COVID-19 are present.

## Introduction

COVID-19 epidemic is caused by SARS-CoV-2 and began in December 2019 in Wuhan, Hubei, China. The virus, which has infected more than 260 million people and killed more than 4.5 million as of December 10, 2021, can cause various conditions, from asymptomatic to lightning-fast respiratory failure (1, 2). Given the rapid community and congregate setting transmission and high pathogenicity, reliable and early identification of SARS-CoV-2 are critical (3). Currently, COVID-19 diagnostic techniques are classified into two categories: (1) methods that evaluate clinical samples directly for virus particles, antigens, or nucleic acids; and (2) serological assays for anti-SARS-CoV-2 antibodies (4). For COVID-19 diagnosis, the reverse transcriptase-polymerase chain reaction (RT-PCR) is the gold standard for sputum, nasopharyngeal swabs, bronchoalveolar lavage fluid, and nasal and nasal oral fluids (5). However, its widespread use is limited by the necessity for expensive laboratory equipment and well-trained laboratory personnel (6). Furthermore, these tests are frequently challenged for being too sensitive since they do not distinguish between live infections and non-viable viral remaining genetic pieces. On the other hand, these diagnostic tests can tell if a disease is present in a person but not define its contagiousness (7).

Recent studies have shown rapid antigen test (RAT) to be a more practical, less costly, and faster technique, especially in the early days following symptoms, although less sensitive (8). Antigen diagnostic assays identify proteins from a live virus in 15–30 min, such as the spike protein, nucleocapsid protein, or both (9). It is cost-effective, easy to use outside of laboratory facilities, requires no experienced workers, and may be used on a wide range of patients. Many of these tests do not need the use of analyzers or readers, making them less costly and more portable (10). Another benefit of employing an antigen test is that it may discover vast numbers of asymptomatic carriers who often migrate from one location to another. This test may also be used as a preliminary screening test before RT-PCR (11). However, despite their excellent specificity, the sensitivity of RAT kits is not as great as other molecular assays (12). Thus, the efficacy of RAT is yet to be investigated comprehensively. Therefore, the current systematic review and meta-analysis were conducted to evaluate the diagnostic accuracy of RAT against RT-PCR methods as the reference standard.

## Methods

This study was conducted and reported according to the PRISMA guidelines (13).

### Search Strategy and Selection Criteria

The MEDLINE/PubMed and Embase were searched for relevant studies published up to March 8 2022. The combination of the following keywords was used: (Sensitivity and Specificity) OR (predictive value) OR (accuracy) AND (COVID-19) OR (SARS-CoV-2). We used a combination of free text and MeSH terms to identify the relevant studies. Studies were included if they used commercial RAT as their index test and RT-PCR as their reference test to detect SARS-CoV-2 and provide sufficient data to compute sensitivity and specificity. Only English studies were included. Duplicate publications, protocols, reviews, conference abstracts, and in-house tests were excluded.

### Extraction of Data

Two reviewers (MA and AFS) designed a data extraction form. These reviewers extracted data from all eligible studies, and consensus resolved differences. The following items were extracted from each article: the name of the first author, year of publication, study location, RT-PCR test, number of confirmed SARS-CoV-2 positive cases, cycle threshold (Ct) value, presence of symptoms, specimen types, and type of antigen tests.

### Quality Assessment

The methodological quality of the studies was assessed using the QUADAS-2 checklist (14). The following items are evaluated in this checklist: Patient selection: describes methods of patient selection; index text: describes the index test and how it was conducted and interpreted; reference standard: describes the reference standard (standard gold test) and how it was conducted and interpreted; flow and timing: describes any patients who did not receive the index tests or reference standard and defines the interval and any interventions between index tests and the reference standard.

### Statistical Analysis

Statistical analyses were performed with Meta-DiSc (version 1.4, Cochrane Colloquium, Barcelona, Spain) software. The pooled sensitivity, specificity, and diagnostic odds ratio (DOR) with 95% confidence intervals between antigen rapid diagnostic tests and the reference standard were assessed. A random-effects model was used to pool the estimated effects. The random-effects model was used because of the estimated heterogeneity of the true effect sizes. Diagnostic accuracy measures [(i.e., the summary receiver operating characteristic (SROC) curve and the summary positive likelihood ratios (PLR), negative likelihood ratios (NLR), and DOR] were calculated.

Sensitivity is the proportion of positive test results among those with the target infection. Specificity is the proportion of negative test results among those without the disease. The PLR measures how frequently a positive test is found in infected vs. non-infected individuals. On the other hand, the NLR measures how likely a negative result is in infected vs. non-infected individuals. Tests with pooled PLR values >10 and a pooled NLR value of <0.1 have the greater discriminating ability (15, 16).

The DOR or the odds of a positive result in infected individuals compared to the odds of a positive result in non-infected individuals. It is calculated according to the formula: DOR = (TP/FN)/(FP/TN). DOR depends significantly on the sensitivity and specificity of a test. A high specificity and sensitivity test with a low rate of false positives and false negatives have high DOR (16).

The area under the curve (AUC) serves as a global measure of test performance; a value of 1 indicates perfect accuracy (16, 17).

Deek's test was used to identify the risk of publication bias based on parametric linear regression methods (18). Subgroup analysis was conducted using several study characteristics separately.

## Results

**Figure 1:**
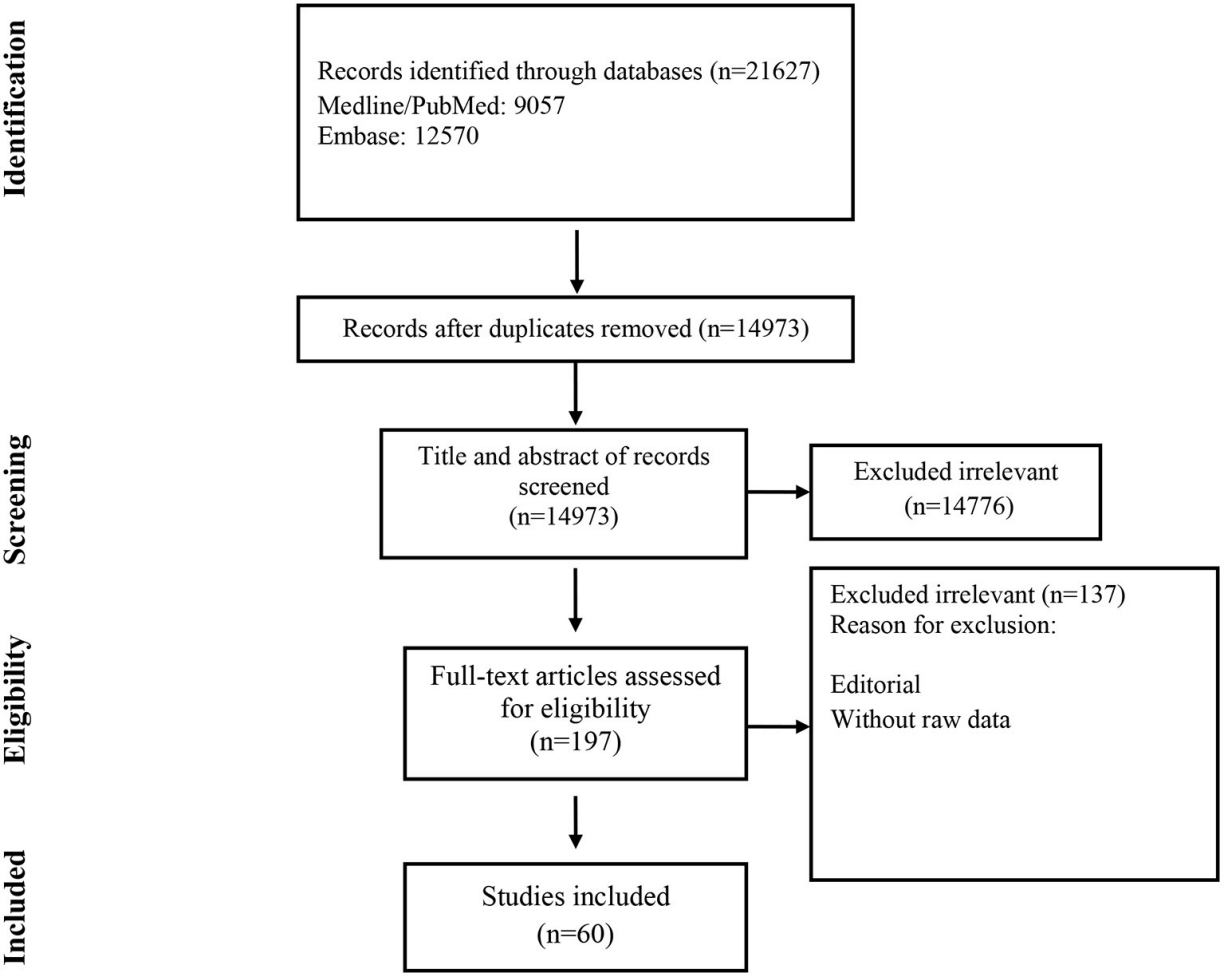
Flow chart of study selection for inclusion in the systematic review and meta-analysis.

Studies included and excluded through the review process are summarized in Figure 1. A total of 21,627 records were found in the initial search; after removing duplicate articles, titles and abstracts of 14,973 references were screened. One hundred ninety-seven articles were selected for a full-text review. Of these, 137 were excluded because they did not present primary data. Finally, 60 were chosen (Table 1) (19–78).

**Table 1:** Characterization of included studies.

**First author**	**Country**	**Sample**	**Rapid antigen test**	**Gene detected by real-time PCR**
James et al. (36)	USA	Nasal swab	Rapid Antigen Test (BinaxNOW)	N gene
McKay et al. (46)	France	Nasopharyngeal swab	Rapid Antigen Test (BinaxNOW)	NR
Prince-Guerra et al. (70)	USA	Respiratory swab	Rapid Antigen Test (BinaxNOW)	NR
Sood et al. (42)	USA	Oral fluid	Rapid Antigen Test (BinaxNOW)	NR
Caputoa et al. (25)	Italy	Nasopharyngeal/oropharyngeal swab	Rapid Antigen Test (Lumipulse G)	NR
Hirotsu et al. (69)	Japan	Nasopharyngeal swab	Rapid Antigen Test (Lumipulse G)	N gene
Gilli et al. (48)	Italy	Nasopharyngeal swab	Rapid Antigen Test (Lumipulse G)	E and N genes
Ishii et al. (34)	Japan	Nasopharyngeal swab	Rapid Antigen Test (Lumipulse G)	NR
Alemany et al. (63)	Spain	Nasopharyngeal swab	Rapid Antigen Test (Panbio)	NR
Akingbaa et al. (53)	South Africa	Nasopharyngeal swab	Rapid Antigen Test (Panbio)	NR
Albert et al. (19)	Spain	Nasopharyngeal swab	Rapid Antigen Test (Panbio)	NR
Berger et al. (23)	Switzerland	Nasopharyngeal swab	Rapid Antigen Test (Panbio)	E gene
Favresse et al. (30)	Belgium	Nasopharyngeal swab	Rapid Antigen Test (Panbio)	E and N genes
Gremmels et al. (67)	Netherlands	Nasopharyngeal swab	Rapid Antigen Test (Panbio)	E and N genes
Jaaskelainen et al. (35)	Finland	Nasopharyngeal swab	Rapid Antigen Test (Panbio)	N gene
Linares et al. (71)	Spain	Nasopharyngeal swab	Rapid Antigen Test (Panbio)	NR
Masiá et al. (40)	Spain	Nasopharyngeal and nasal swab	Rapid Antigen Test (Panbio)	E and N genes
Matsuda et al. (29)	Brazil	Nasopharyngeal/oropharyngeal swab	Rapid Antigen Test (Panbio)	E and N genes
Nsoga et al. (50)	Switzerland	Nasopharyngeal/oropharyngeal swab	Rapid Antigen Test (Panbio)	E gene
Perez-García et al. (31)	Spain	Nasopharyngeal/oropharyngeal swab	Rapid Antigen Test (Panbio)	E, S and N genes
Strömer et al. (21)	Germany	Nasopharyngeal swab	Rapid Antigen Test (Panbio)	E and N genes
Torres et al. (47)	Spain	Nasopharyngeal swab	Rapid Antigen Test (Panbio)	N gene
Villaverde et al. (52)	Spain	Nasopharyngeal swab	Rapid Antigen Test (Panbio)	E gene
Ciotti et al. (27)	Italy	Nasopharyngeal swab	Rapid Antigen Test (Respi-Strip)	E and N genes
Mertens et al. (72)	Belgium	Nasopharyngeal/oropharyngeal swab	Rapid Antigen Test (Respi-Strip)	E gene
Scohy et al. (74)	Belgium	Nasopharyngeal swab	Rapid Antigen Test (Respi-Strip)	NR
Lambert-Niclot et al. (77)	France	Nasopharyngeal swab	Rapid Antigen Test (Respi-Strip)	E gene
Noerz et al. (60)	Germany	Nasopharyngeal/oropharyngeal swab	Rapid Antigen Test (Roche Diagnostics)	E gene
Baro et al. (22)	Spain	Nasopharyngeal swab	Rapid Antigen Test (Roche Diagnostics)	NR
Kohmer et al. (37)	Germany	Nasopharyngeal swab	Rapid Antigen Test (Roche Diagnostics)	NR
Kruttgen et al. (38)	Germany	Nasopharyngeal swab	Rapid Antigen Test (Roche Diagnostics)	NR
Lgloi et al. (39)	Netherlands	Nasopharyngeal/oropharyngeal swab	Rapid Antigen Test (Roche Diagnostics)	NR
Osterman et al. (20)	Germany	Nasopharyngeal/oropharyngeal swab	Rapid Antigen Test (Roche Diagnostics)	N gene
Salvagno et al. (32)	Italy	Nasopharyngeal swab	Rapid Antigen Test (Roche Diagnostics)	E and N genes
Cerutti et al. (65)	Italy	Nasopharyngeal swab	Rapid Antigen Test (SD Biosensor)	NR
Chaimayo et al. (66)	Thailand	Nasopharyngeal and throat swab	Rapid Antigen Test (SD Biosensor)	E and N genes
Gupta et al. (68)	India	Nasopharyngeal swab and sputum	Rapid Antigen Test (SD Biosensor)	NR
Kannian et al. (54)	India	Whole mouth fluid	Rapid Antigen Test (SD Biosensor)	NR
Bruzzonea et al. (24)	Italy	Nasopharyngeal swab	Rapid Antigen Test (SD Biosensor)	N gene
Caruana et al. (59)	Switzerland	Nasopharyngeal swab	Rapid Antigen Test (SD Biosensor)	NR
Homza et al. (33)	Czech republic	Nasopharyngeal swab	Rapid Antigen Test (SD Biosensor)	NR
Lindner et al. (73)	Germany	Nasopharyngeal/oropharyngeal swab	Rapid Antigen Test (SD Biosensor)	NR
Liotti et al. (78)	Italy	Nasopharyngeal swab	Rapid Antigen Test (SD Biosensor)	NR
Peñaa et al. (56)	Chile	Nasopharyngeal swab	Rapid Antigen Test (SD Biosensor)	S and N genes
Peña-Rodríguez et al. (41)	Mexico	Nasopharyngeal/oropharyngeal swab	Rapid Antigen Test (SD Biosensor)	N gene
Turcato et al. (61)	Italy	Nasopharyngeal swab	Rapid Antigen Test (SD Biosensor)	NR
Courtellemont et al. (28)	France	Oropharyngeal and/or saliva swab	Rapid Antigen Test (VIRO)	E, S and N genes
Houston et al. (49)	UK	Nasopharyngeal swab	Rapid Antigen Test (Innova SARS-CoV-2)	NR
Wagenhauser et al. (58)	Germany	Oropharyngel swab	Rapid Antigen Test (NADAL)	S gene
Mboumba Bouassa et al. (44)	France	Nasopharyngeal swab	Rapid Antigen Test (Sienna)	N gene
Takeuchi et al. (57)	Japan	Nasopharyngeal/oropharyngeal swab	Rapid Antigen Test (QuickNavi)	N gene
Pekosz et al. (64)	USA	Respiratory swab	Rapid Antigen Test (BD Life Sciences)	NR
Weitzel et al. (76)	Chile	Nasopharyngeal/oropharyngeal swab	Rapid Antigen Test (Biocredit)	NR
Häuser et al. (45)	Germany	Nasopharyngeal swab	Rapid Antigen Test (DiaSorin)	NR
Caruana et al. (26)	Switzerland	Nasopharyngeal/oropharyngeal swab	Rapid Antigen Test (Exdia)	N gene
Thakur et al. (43)	India	Nasopharyngeal swab	Rapid Antigen Test (PathoCatch)	E gene
Micocci et al. (55)	UK	Nasopharyngeal swab	Rapid Antigen Test (LumiraDx)	NR
Osmanodja et al. (51)	Germany	Nasopharyngeal/oropharyngeal swab	Rapid Antigen Test (Dräger Antigen Test)	E gene
Shrestha et al. (75)	Nepal	Nasopharyngeal swab	Rapid Antigen Test (Biocredit)	NR
Young et al. (62)	UK	Nasopharyngeal swab	Rapid Antigen Test (Lateral Flow)	NR

*NR, Not reported*.

Of these, 148 were excluded because they did not present primary data (13, 19–94); or the Ag-RDT was not commercially available (16), 132–164, leaving 133 studies to be included in the systematic review.

A total of 43,034 samples (8,360 with and 34,674 without COVID-19) were investigated. The included studies came from different countries, with the majority from Germany (*n* = 9), followed by Spain and Italy (*n* = 8). Participants in the included studies varied from being either symptomatic only (*n* = 13), asymptomatic only (*n* = 9), or a mix of both (*n* = 24). The included studies had either adults only or participants of all ages. Three studies evaluated the diagnostic performance of antigen tests with nasal/oral swab specimens, and 52 the accuracy of antigen tests with nasopharyngeal swab specimens. Twenty-seven studies provided Ct values of positive RT-PCRs. The investigated commercial RAT was Panbio, SD Biosensor, Roche, COVID-19 Ag Respi-Strip, LUMIPULSE, and BinaxNOW. All RAT detected nucleocapsid or spike proteins.

### Quality of Including Studies

Forty-five studies were judged to have a high risk of bias in the patient selection domain. Based on the QUADAS 2 tool, in these studies, patient selection methods were not fully described. Furthermore, a high risk of bias was found in the domain of the index tests in 27 studies. In thesis studies, it was not clear whether the index test results were interpreted without knowledge of the results of the reference standard. All studies underwent a reference standard and were judged to have a low risk of bias in the flow and timing domains (Table 2).

**Table 2:** Quality assessment of included studies.

**Study**	**Risk of bias**				**Applicability concerns**		
	**Patient selection**	**Index test**	**Reference standard**	**Flow and timing**	**Patient selection**	**Index test**	**Reference standard**
Albert	Low risk	Low risk	Low risk	Low risk	Low risk	Low risk	Low risk
Osterman	High risk	Low risk	Low risk	Low risk	Low risk	Low risk	Low risk
Strömer	High risk	Low risk	Low risk	Low risk	Low risk	Low risk	Low risk
Baro	High risk	Low risk	Low risk	Low risk	Low risk	Low risk	Low risk
Berger	High risk	Low risk	Low risk	Low risk	Low risk	Low risk	Low risk
Caruana	High risk	Low risk	Low risk	Low risk	Low risk	Low risk	Low risk
Ciotti	High risk	Low risk	Low risk	Low risk	Low risk	Low risk	Low risk
Courtellemont	Low risk	Low risk	Low risk	Low risk	Low risk	Low risk	Low risk
Matsuda	Low risk	Low risk	Low risk	Low risk	Low risk	Low risk	Low risk
Favresse	Low risk	Low risk	Low risk	Low risk	Low risk	Low risk	Low risk
Perez-García	Low risk	Low risk	Low risk	Low risk	Low risk	Low risk	Low risk
Salvagno	Low risk	Low risk	Low risk	Low risk	Low risk	Low risk	Low risk
Ishii	High risk	Low risk	Low risk	Low risk	Low risk	Low risk	Low risk
Kohmer	High risk	Low risk	Low risk	Low risk	Low risk	Low risk	Low risk
Kruttgen	High risk	Low risk	Low risk	Low risk	Low risk	Low risk	Low risk
Lgloi	Low risk	Low risk	Low risk	Low risk	Low risk	Low risk	Low risk
Peña-Rodríguez	High risk	Low risk	Low risk	Low risk	Low risk	Low risk	Low risk
Thakur	High risk	Low risk	Low risk	Low risk	Low risk	Low risk	Low risk
Bouassa	High risk	Low risk	Low risk	Low risk	Low risk	Low risk	Low risk
Häuser	Low risk	Low risk	Low risk	Low risk	Low risk	Low risk	Low risk
McKay	High risk	Low risk	Low risk	Low risk	Low risk	Low risk	Low risk
Gilli	High risk	Low risk	Low risk	Low risk	Low risk	Low risk	Low risk
Osmanodja	Low risk	Low risk	Low risk	Low risk	Low risk	Low risk	Low risk
Micocci	High risk	Low risk	Low risk	Low risk	Low risk	Low risk	Low risk
Peñaa	Low risk	Low risk	Low risk	Low risk	Low risk	Low risk	Low risk
Caruana	High risk	Low risk	Low risk	Low risk	Low risk	Low risk	Low risk
Noerz	High risk	Low risk	Low risk	Low risk	Low risk	Low risk	Low risk
Chaimayo	High risk	Low risk	Low risk	Low risk	Low risk	Low risk	Low risk
Gremmels	High risk	Low risk	Low risk	Low risk	Low risk	Low risk	Low risk
Mertens	High risk	Low risk	Low risk	Low risk	Low risk	Low risk	Low risk
Lindner	Low risk	Low risk	Low risk	Low risk	Low risk	Low risk	Low risk
Scohy	High risk	Low risk	Low risk	Low risk	Low risk	Low risk	Low risk
Lambert-Niclot	High risk	Low risk	Low risk	Low risk	Low risk	Low risk	Low risk
Bruzzonea	High risk	High risk	Low risk	Low risk	Low risk	Low risk	Low risk
Caputoa	High risk	High risk	Low risk	Low risk	Low risk	Low risk	Low risk
Homza	High risk	High risk	Low risk	Low risk	Low risk	Low risk	Low risk
Jaaskelainen	High risk	High risk	Low risk	Low risk	Low risk	Low risk	Low risk
James	High risk	High risk	Low risk	Low risk	Low risk	Low risk	Low risk
Masiá	High risk	High risk	Low risk	Low risk	Low risk	Low risk	Low risk
Sood	High risk	High risk	Low risk	Low risk	Low risk	Low risk	Low risk
Torres	High risk	High risk	Low risk	Low risk	Low risk	Low risk	Low risk
Houston	High risk	High risk	Low risk	Low risk	Low risk	Low risk	Low risk
Nsoga	Low risk	High risk	Low risk	Low risk	Low risk	Low risk	Low risk
Villaverde	Low risk	High risk	Low risk	Low risk	Low risk	Low risk	Low risk
Akingbaa	High risk	High risk	Low risk	Low risk	Low risk	Low risk	Low risk
Kannian	High risk	High risk	Low risk	Low risk	Low risk	Low risk	Low risk
Takeuchi	High risk	High risk	Low risk	Low risk	Low risk	Low risk	Low risk
Wagenhauser	High risk	High risk	Low risk	Low risk	Low risk	Low risk	Low risk
Turcato	High risk	High risk	Low risk	Low risk	Low risk	Low risk	Low risk
Young	High risk	High risk	Low risk	Low risk	Low risk	Low risk	Low risk
Alemany	High risk	High risk	Low risk	Low risk	Low risk	Low risk	Low risk
Pekosz	Low risk	High risk	Low risk	Low risk	Low risk	Low risk	Low risk
Cerutti	Low risk	High risk	Low risk	Low risk	Low risk	Low risk	Low risk
Gupta	Low risk	High risk	Low risk	Low risk	Low risk	Low risk	Low risk
Hirotsu	High risk	High risk	Low risk	Low risk	Low risk	Low risk	Low risk
Prince-Guerra	High risk	High risk	Low risk	Low risk	Low risk	Low risk	Low risk
Linares	High risk	High risk	Low risk	Low risk	Low risk	Low risk	Low risk
Shrestha	High risk	High risk	Low risk	Low risk	Low risk	Low risk	Low risk
Weitzel	High risk	High risk	Low risk	Low risk	Low risk	Low risk	Low risk
Liotti	High risk	High risk	Low risk	Low risk	Low risk	Low risk	Low risk

### Diagnostic Accuracy of Rapid Antigen Tests Against Reference Test

**Figure 2:**
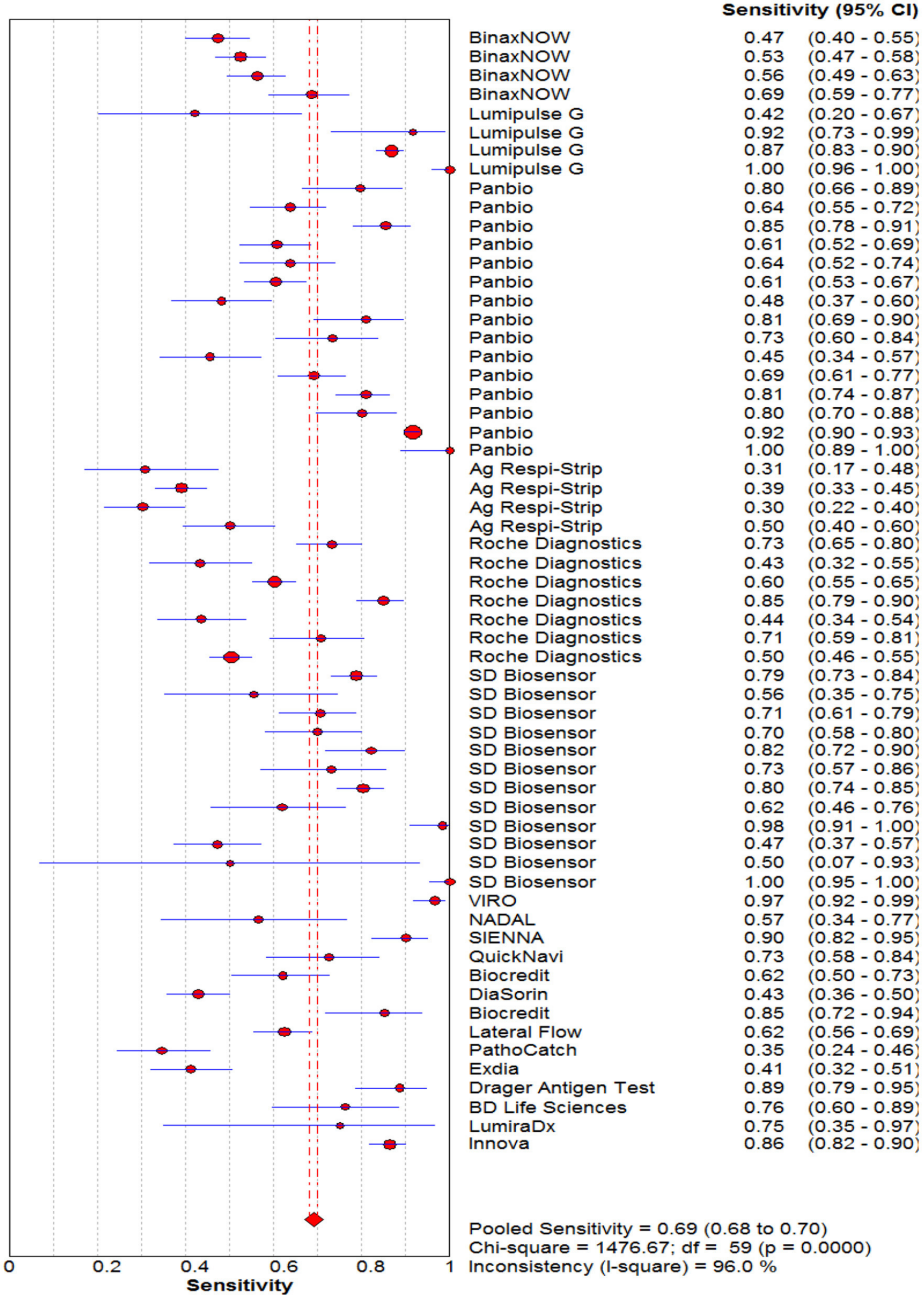
Forest plot of pooled sensitivity of rapid antigen tests for the diagnosis of COVID-19. The point estimates of sensitivity from each study are indicated as a circle and a 95% confidence interval is shown with a horizontal line.

**Figure 3:**
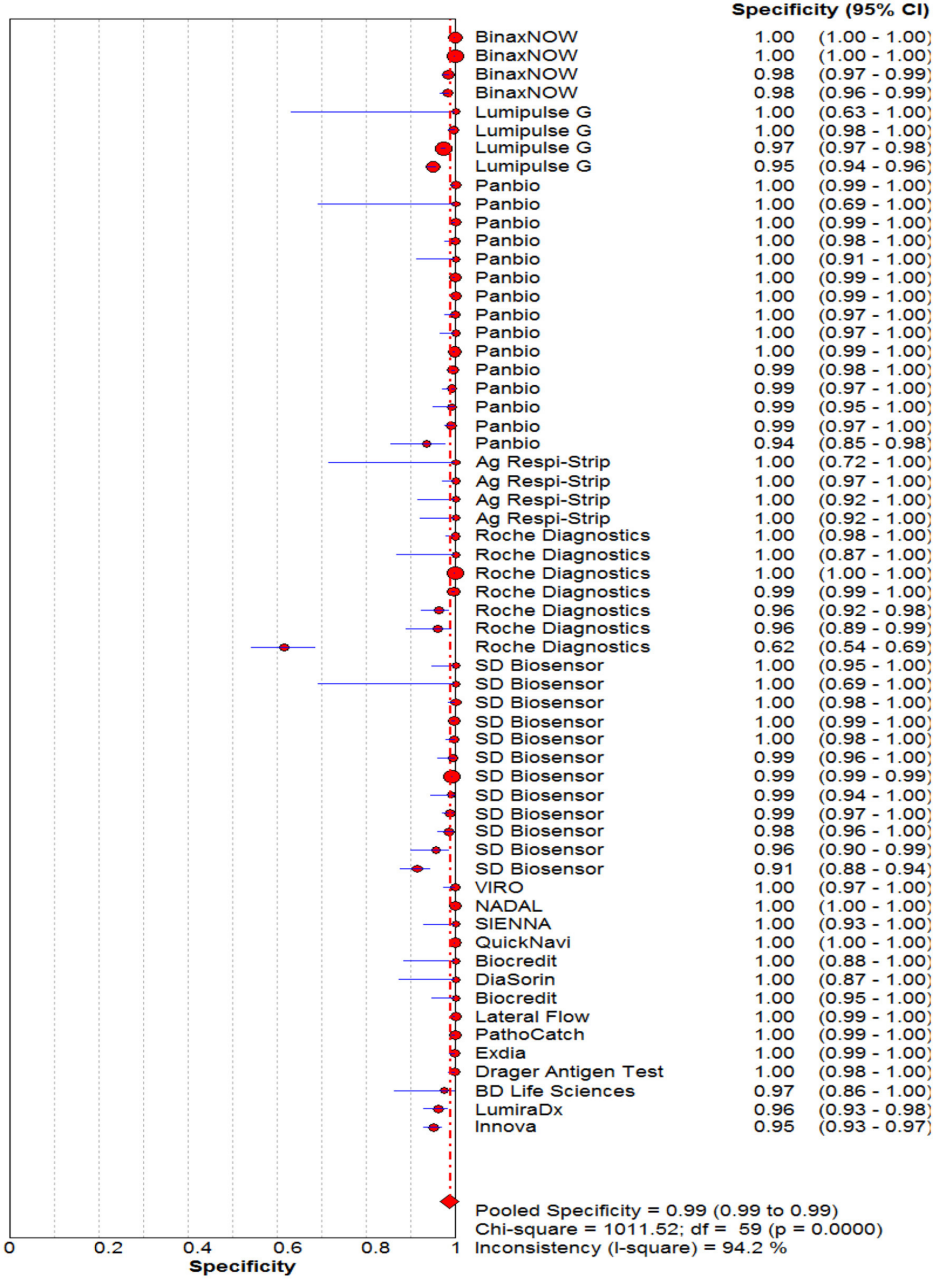
Forest plot of pooled specificity of rapid antigen tests for the diagnosis of COVID-19. The point estimates of specificity from each study are indicated as a circle and a 95% confidence interval is shown with a horizontal line.

**Figure 4:**
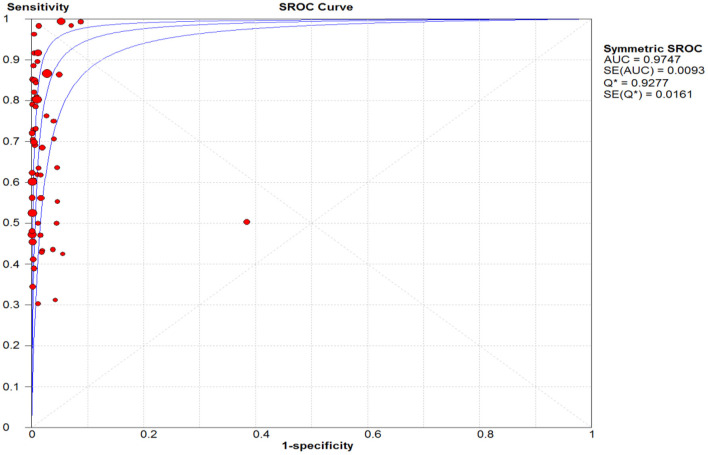
Summary receiver operating characteristic (SROC) plot. The area under the curve (AUC), acts as an overall measure for test performance. Particularly, when AUC would be between 0.9 and 1, the accuracy is high. AUC was 0.98 in this report which represented a high level of accuracy.

The pooled sensitivity and specificity of the RAT were 69% (95% CI: 68–70) and 99% (95% CI: 99–99) (Figures 2, 3). The PLR, NLR, DOR, and the AUC estimates were found to be 72 (95% CI: 44–119), 0.30 (95% CI: 0.26–0.36), 316 (95% CI: 167–590), and 97%, respectively. The AUC estimates in this report also represented a high level of test accuracy (Figure 4). Deek's test result indicated no likelihood for publication bias (*P* > 0.05).

### Subgroup Analyses

The sensitivity for each subgroup was lower than the specificity (Table 3). The sensitivity of RAT was slightly higher in symptomatic (65%) than asymptomatic patients (64%). Kits from different manufacturers exhibited various sensitivity. Lumipulse showed the highest sensitivity (87%) followed by SD Biosensor (76%), Panbio (75%), Roche (60%), BinaxNOW (57%) and Respi-Strip (39%). The sensitivity of the nasopharyngeal swab was higher (70%) than that where throat or saliva swabs were used (52%). The sensitivity of RAT kits ranged from 65 to 71% when Ct values were 20–31. The RAT kits had a similar sensitivity based on the antigen detection technology (i.e., immunochromatography and chemiluminescent immunoassay). The pooled sensitivity for Ct value ≤ 25 was markedly better, at 71.0%, compared to the group with Ct value >26, at 67.0%.

**Table 3:** Pooled sensitivity and specificity among subgroups of studies.

**Subgroups**	**No. of study**	**No. of tested individuals**	**Sensitivity**	**Specificity**
			**(95 % CI)**	**(95 % CI)**
**Presence of symptoms**				
Symptomatic	13 studies	9081	65.0 (63.0–67.0)	98.0 (97.0–99.0)
Asymptomatic	9 studies	3696	64.0 (61.0−67.0)	98.0 (97.0–99.0)
**Antigen tests**				
Lumipulse G	4 studies	6517	87.0 (85.0–90.0)	97.0 (96.0–98.0)
COVID-19 Ag Respi-Strip	4 studies	736	39.0 (34.0–43.0)	100 (98.0–100.0)
SD Biosensor	12 studies	6887	76.0 (73.0–78.0)	99.0 (95.0–100)
Panbio	15 studies	12577	75.0 (73.0–76.0)	100 (100–100)
BinaxNOW	4 studies	4725	57.0 (53.0–60.0)	99.0 (99.0–100)
Roche Diagnostics	7 studies	5601	60.0 (58.0–63.0)	98.0 (97.0–98.0)
**Antigen detection technology**				
Immunochromatography	48 studies	30128	72.0 (71.0–73.0)	99.0 (99.0–99.0)
Chemiluminescent immunoassay	6 studies	9879	72.0 (69.0–74.0)	98.0 (97.0–98.0)
**Specimen types**				
Nasopharyngeal Swab	52 studies	30251	70.0 (69.0–71.0)	98.0 (98.0–98.0)
Other (Nasal and oral)	3 studies	3150	52.0 (48.0–57.0)	100 (99.0–100)
**Mean Ct values**				
Ct value ≤ 25	15 studies	7540	71.0 (70.0–73.0)	98.0 (98.0–98.0)
Ct value >26	9 studies	2988	67.0 (64.0–70.0)	98.0 (98.0–99.0)

## Discussion

Diagnostic testing for SARS-CoV-2 is essential for the overall COVID-19 preventive and control plan. With the number of COVID-19 cases and mortalities increasing worldwide, it is more important than ever to look into the usefulness of existing diagnostic tests and the optimal settings to achieve the most accuracy and consistency (4).

RT-PCR has been accepted as the gold standard for SARS-CoV-2 infection diagnosis. Despite its high sensitivity and specificity, this method is expensive and needs well-equipped facilities (79). Moreover, the reporting of RT-PCR data may take longer than expected in many cases owing to large sample numbers and a lack of technical assistance, resulting in delayed patient care and outbreak control (80). Consequently, a focus on using RAT kits was required to bridge diagnostic gaps. RAT available on the market is steadily rising (81).

RATs are straightforward to conduct and interpret at the point of care by minimally educated health professionals (82, 83).

We summarized the data from 60 studies evaluating the accuracy of RAT. The sensitivity and specificity were assessed using a reliable reference standard test. The pooled estimates of sensitivity and specificity of the RAT against RT-PCR were 69 and 99%, respectively.

Similarly, Lee et al. (84) computed a sensitivity of 68% and a specificity of 99% for 24 studies focused on RAT (84). The meta-analysis of Wang et al. (85) showed a sensitivity of 79% and a specificity of 100%, pooling 14 studies (85). Likewise, according to the meta-analysis performed by Brummer et al., the sensitivity and specificity of RAT were 71.2 and 98.9%, respectively (81). In a study by Chen et al., the diagnostic accuracy of RAT for SARS-CoV-2 in community participants was assessed. The overall sensitivity and specificity were 82 and 100%, respectively (86).

The World Health Organization (WHO) recommends that a RAT kit reach a minimum performance criterion of at least 80% sensitivity and 97% specificity (87). Furthermore, RAT findings will be most acceptable in places where community transmission is continuous (5% test positive rate), according to WHO standards (87). RAT positive predictive value is poor when there is no or low transmission (many false positives). RT-PCR is better as a first-line diagnostic tool than confirming positive RAT (88).

In our meta-analysis, sensitivity below 80% and high specificity were found. Sensitivity differences of the mentioned meta-analyses may be related to the characteristics of study participants, including whether patients are symptomatic or asymptomatic and the time of sampling after the onset of symptoms. The results of the current meta-analysis support the statement of the Infectious Diseases Society of America (IDSA) guidelines on the correlation between RAT sensitivity and viral load, symptoms, and the timing of the test (89).

Similar to a previous study, low Ct values, the RT-PCR correlate for high virus concentration, resulted in significantly higher RAT sensitivity (90, 91).

RAT also showed higher sensitivity in symptomatic patients than asymptomatic patients (pooled sensitivity 65 vs. 64%), which is to be expected given that samples from patients with symptoms have been shown to contain the highest virus concentrations (90). similarly, studies that enrolled symptomatic patients showed a lower range of Ct values than studies enrolling asymptomatic patients (90–93).

Considering the epidemiological context, clinical history, and available testing funds, clinical decision-making should be used to determine whether negative RAT results necessitate confirmatory testing with RT-PCR or repeat testing with RAT (within 48 h) if RT-PCR assay is not available (94). Owing to the RAT sensitivity (69%), these tests should be used in the initial screening, contact tracing, and monitoring of the outbreak in different countries (87).

There are some limitations. First, we could not assess the correlation between sample conditions (such as storage or transportation) and the sensitivity of RAT. Second, the potential influence of different genetic and structural mutations of SARS-CoV-2 could not be evaluated because of the limited available information. Variants of SARS-CoV-2 may be differently detected by RAT. Finally, the sensitivity of RAT may differ depending on the manufacturer and the country where the kits are produced.

In conclusion, the present study showed that RAT is recommended mainly for the early detection of patients with presumed COVID-19, especially in countries with limited resources and laboratory equipment. However, negative RAT samples should be confirmed by molecular tests, mainly in the presence of COVID-19 symptoms.

## Data Availability Statement

The original contributions presented in the study are included in the article/supplementary material, further inquiries can be directed to the corresponding authors.

## Author Contributions

All authors participated in the drafting and revision of the manuscript, as well as in the approval of the final version.

## Conflict of Interest

The authors declare that the research was conducted in the absence of any commercial or financial relationships that could be construed as a potential conflict of interest.

## Publisher's Note

All claims expressed in this article are solely those of the authors and do not necessarily represent those of their affiliated organizations, or those of the publisher, the editors and the reviewers. Any product that may be evaluated in this article, or claim that may be made by its manufacturer, is not guaranteed or endorsed by the publisher.
